# Supplementary material for: Epidemiological and phylogenetic analysis reveals Flavobacteriaceae as potential ancestral source of tigecycline resistance gene tet(X)
Source: Nat Commun. 2020 Sep 16;11:4648. doi: 10.1038/s41467-020-18475-9 (PMC7494873; doi:10.1038/s41467-020-18475-9)
Supplement: Supplementary file 1 — Supplementary Information [file 41467_2020_18475_MOESM1_ESM.pdf]

Supplementary Materials

Epidemiological and phylogenetic analysis reveals Flavobacteriaceae as potential ancestral source of tigecycline resistance gene *tet*(X)

Rong Zhang[1#], Ning Dong[2#], Zhangqi Shen[3#], Yu Zeng[1], Jiauyue Lu[1], Congcong Liu[1], Hongwei Zhou[1], Yanyan Hu[1], Qiaoling Sun[1], Qipeng Cheng[2,4], Lingbing Shu[1], Jiachang Cai[1], Edward Wai-Chi Chan[4], Gongxiang Chen[1*], Sheng Chen[2*]

[1]Department of Clinical Laboratory, Second Affiliated Hospital of Zhejiang University, School of Medicine, Zhejiang, Hangzhou, China;

[2]Department of Infectious Diseases and Public Health, Jockey Club College of Veterinary Medicine and Life Sciences, City University of Hong Kong, Kowloon, Hong Kong

[3] Beijing Advanced Innovation Center for Food Nutrition and Human Health, College of Veterinary Medicine, China Agricultural University, Beijing, China

[4]State Key Lab of Chemical Biology and Drug Discovery, Department of Applied Biology and Chemical Technology, The Hong Kong Polytechnic University, Hung Hom, China

# These authors contributed equally: Rong Zhang, Ning Dong, Zhangqi Shen

* Corresponding authors. Correspondence to Sheng Chen, Email: shechen@cityu.edu.hk; or Gongxiang Chen, Email: chengongxiang@zju.edu.cn

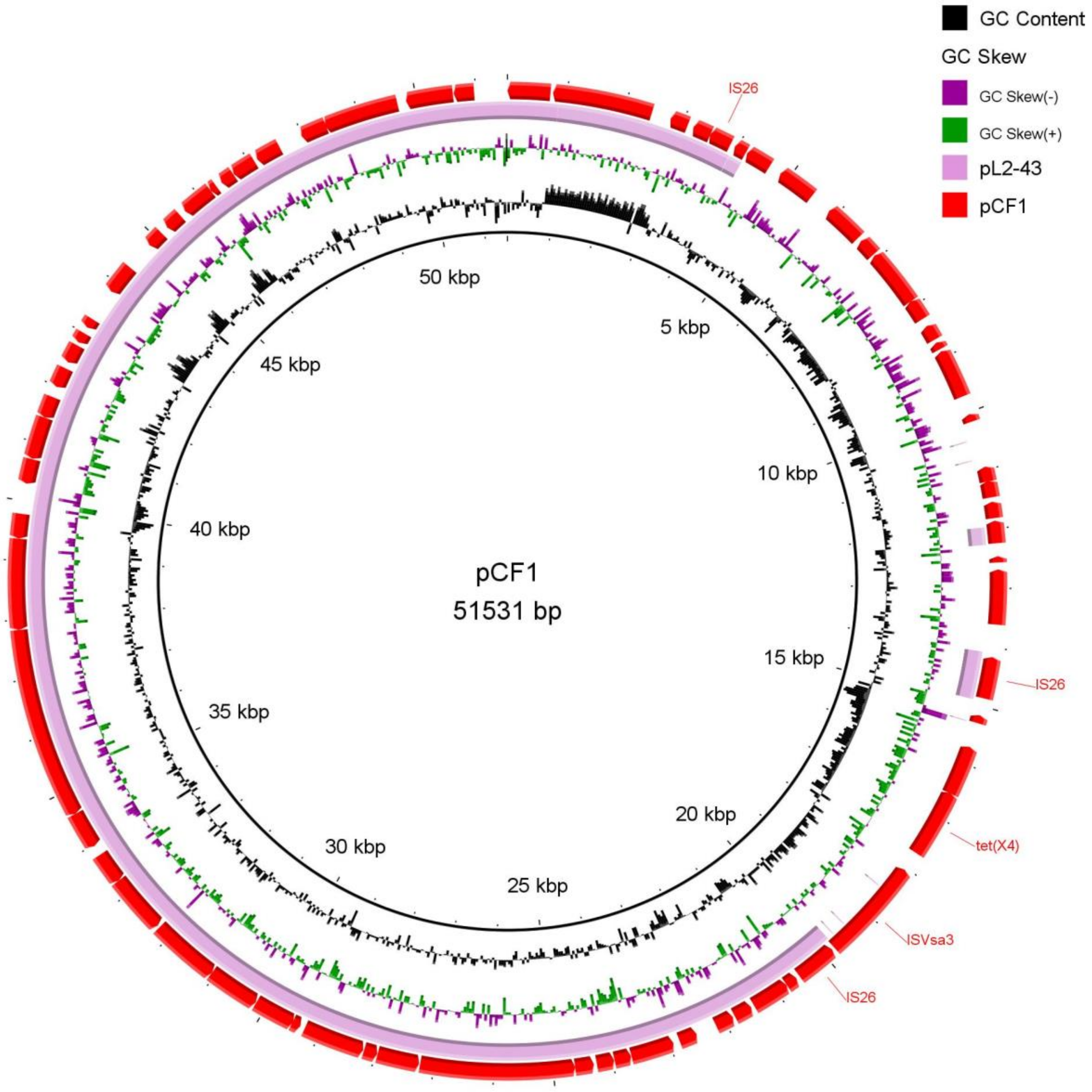

Supplementary Figure 1. Circular map of plasmid pCF1. The inner circle is plasmid pL2-43 (Accession: KJ484641) isolated from an *E. coli* isolate. Insertion sequences and antimicrobial resistance genes are labelled with red fonts.

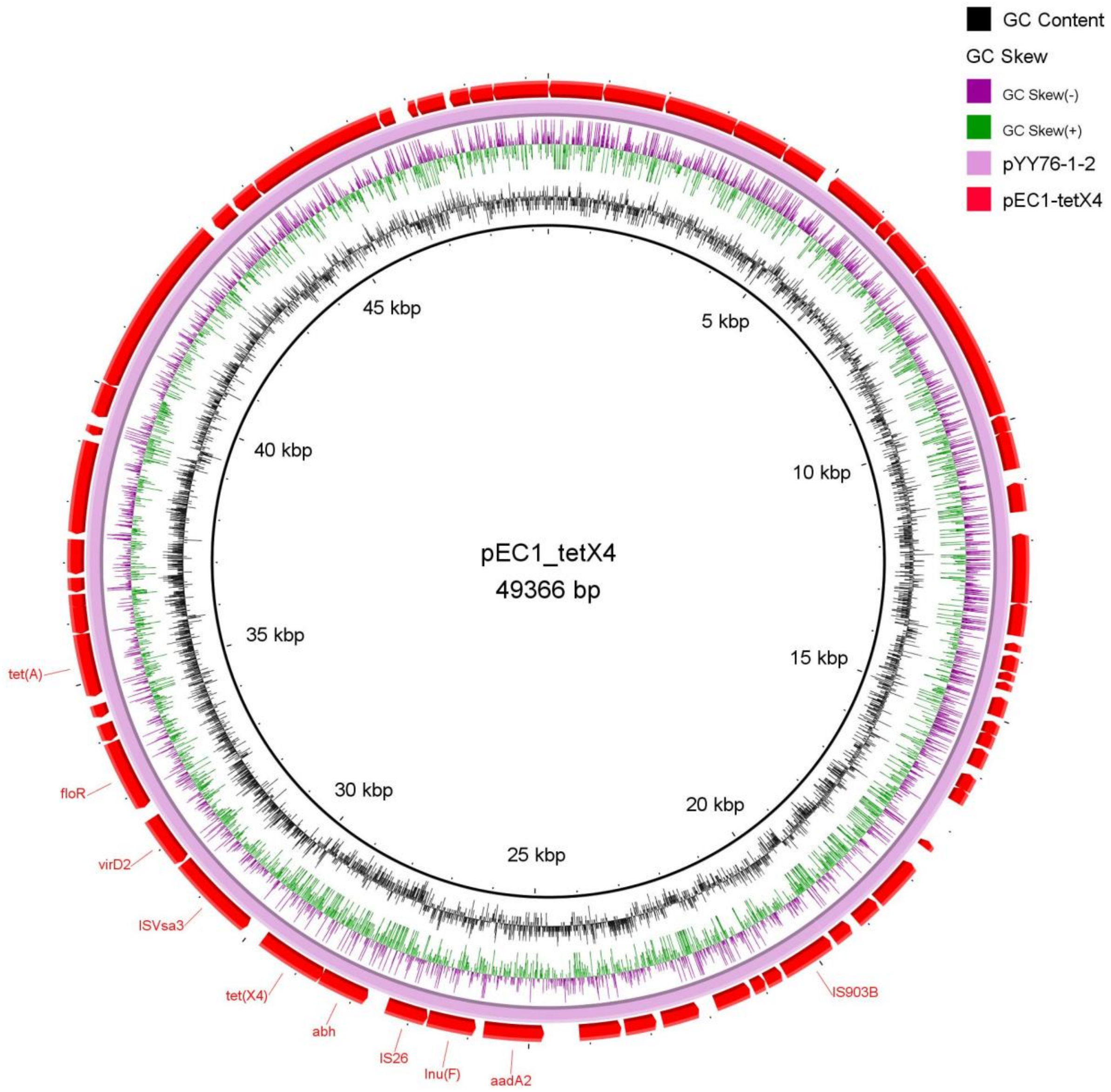

Supplementary Figure 2. Circular map of plasmid pEC1_tetX4. The inner circle is plasmid pYY76-1-2 (accession: CP040929) isolated from an *E. coli* isolate. Insertion sequences and antimicrobial resistance genes are labelled with red fonts.

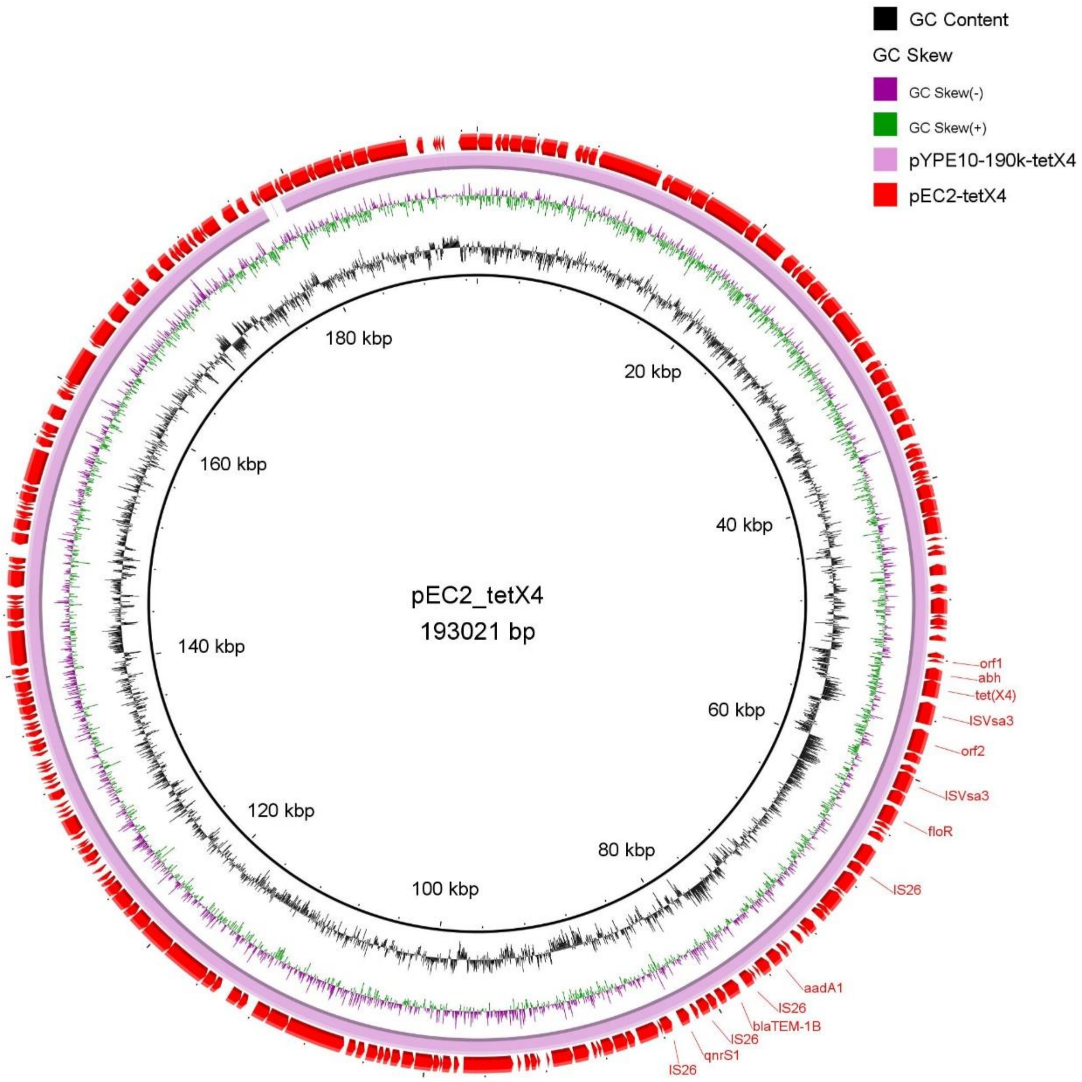

Supplementary Figure 3. Circular map of plasmid pEC2_tetX4. The inner circle is plasmid pYPE10-190k-tetX4 (accession: CP041449) isolated from an *E. coli* isolate. Insertion sequences and antimicrobial resistance genes are labelled with red fonts.

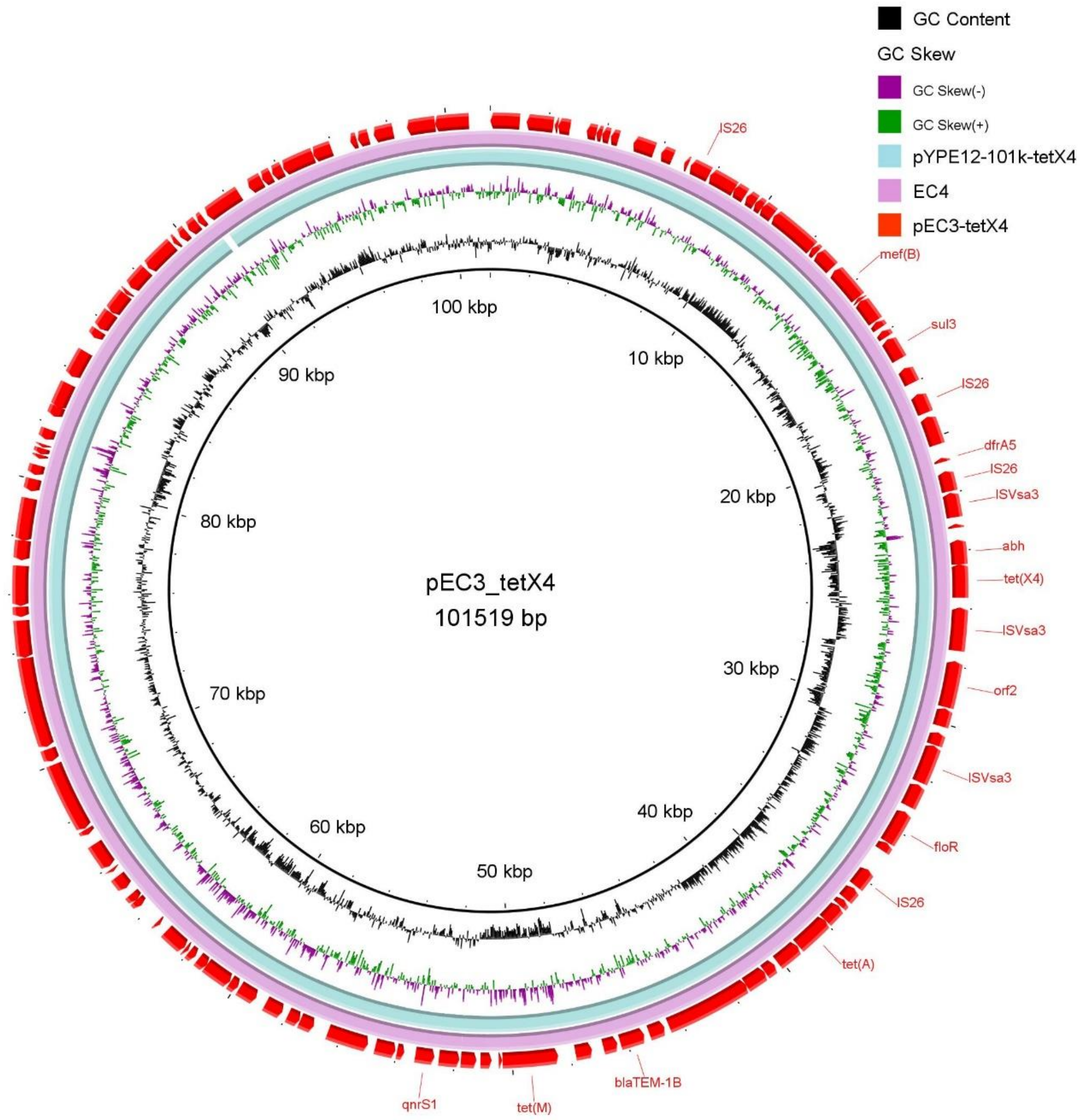

Supplementary Figure 4. Circular map of plasmid pEC3_tetX4. The inner circle is plasmid pYPE12-101k-tetX4 (accession: CP041443) isolated from an *E. coli* isolate. Insertion sequences and antimicrobial resistance genes are labelled with red fonts.

Supplementary Table 1. Primers used in this study

gene	Primer name	Primer sequence	Product size	purpose	Reference
tet(X)	tetXF	TTAGCCTTACCAATGGGTGT	243 bp	*tet*(X)-positive bacteria screening	[1]
	tetXR	CAAATCTGCTGTTTCACTCG			
tet(X3)	tetX3F	CAGGACAGAAACAGCGTTGC	179 bp	gene expression level analysis	[2]
	tetX3R	GCAGCATCGCCAATCATTGT			
tet(X5)-variant	tetX5VF	TGCCTTGATATTGTCGGATAA	113 bp	gene expression level analysis	this study
	tetX5VR	ATTCTGCCTGTGCTTCTC			

References

1 Bartha, N. A., Sóki, J., Urbán, E. & Nagy, E. Investigation of the prevalence of tetQ, tetX and tetX1 genes in Bacteroides strains with elevated tigecycline minimum inhibitory concentrations. *International journal of antimicrobial agents* **38**, 522-525 (2011).

2 Li, Y., Shen, Z., Ding, S. & Wang, S. A TaqMan-based multiplex real-time PCR assay for the rapid detection of tigecycline resistance genes from bacteria, faeces and environmental samples. *BMC microbiology* **20**, 1-7 (2020).
