# Supplementary material for: Epidemiological and phylogenetic analysis reveals Flavobacteriaceae as potential ancestral source of tigecycline resistance gene tet(X)
Source: Nat Commun. 2020 Sep 16;11:4648. doi: 10.1038/s41467-020-18475-9 (PMC7494873; doi:10.1038/s41467-020-18475-9)
Supplement: Supplementary file 3 — Reporting Summary [file 41467_2020_18475_MOESM3_ESM.pdf]

nature research

Corresponding author(s): Sheng Chen; Gongxiang Chen

Last updated by author(s): 2020 July 30

Reporting Summary

Nature Research wishes to improve the reproducibility of the work that we publish. This form provides structure for consistency and transparency in reporting. For further information on Nature Research policies, see our Editorial Policies and the Editorial Policy Checklist.

Statistics

For all statistical analyses, confirm that the following items are present in the figure legend, table legend, main text, or Methods section.

n/a	Confirmed	
☐	☒	The exact sample size (*n*) for each experimental group/condition, given as a discrete number and unit of measurement
☐	☒	A statement on whether measurements were taken from distinct samples or whether the same sample was measured repeatedly
☒	☐	The statistical test(s) used AND whether they are one- or two-sided<br>*Only common tests should be described solely by name; describe more complex techniques in the Methods section.*
☒	☐	A description of all covariates tested
☒	☐	A description of any assumptions or corrections, such as tests of normality and adjustment for multiple comparisons
☐	☒	A full description of the statistical parameters including central tendency (e.g. means) or other basic estimates (e.g. regression coefficient) AND variation (e.g. standard deviation) or associated estimates of uncertainty (e.g. confidence intervals)
☒	☐	For null hypothesis testing, the test statistic (e.g. *F*, *t*, *r*) with confidence intervals, effect sizes, degrees of freedom and *P* value noted<br>*Give P values as exact values whenever suitable.*
☒	☐	For Bayesian analysis, information on the choice of priors and Markov chain Monte Carlo settings
☒	☐	For hierarchical and complex designs, identification of the appropriate level for tests and full reporting of outcomes
☒	☐	Estimates of effect sizes (e.g. Cohen's *d*, Pearson's *r*), indicating how they were calculated

Our web collection on statistics for biologists contains articles on many of the points above.

Software and code

Policy information about availability of computer code

Data collection	To identify sequence homologous to tet(X) and its orthologues, a BLASTp v.2.10.0 search was performed using the amino acid sequences of TetX to TetX5 as query sequences.
Data analysis	Draft genome assembly was conducted with SPAdes v3.12.0. Hybrid assembly of Illumina and nanopore sequencing reads was constructed using Unicycler v 0.3.0. Genome sequences were annotated using RAST v2.0 with manual editing. Multilocus sequence typing (MLST) of all Enterobacteriaceae isolates was conducted using MLST v2.11. Acquired antibiotic resistance genes were identified by ResFinder 2.1. Heatmap of antimicrobial resistance genes was plotted using Genesis v1.7.7. Plasmid replicons were analyzed using PlasmidFinder 2.1. Insertion sequences (ISs) were identified using ISfinder v2.0 (https://isfinder.biotoul.fr/about.php). 16S rRNA genes of all isolates were obtained using Barrnap v0.9. Integrative and conjugative elements were predicted using ICEberg2. A neighbor-joining tree of all tet(X)-positive isolates based on the alignment of the 16S rRNA gene sequences was conducted using the CLC Genomics Workbench v9 (Qiagen, CA, USA) using a bootstrap value of 1000. GC-content of all genome assemblies were calculated using CLC Genomics Workbench v9.<br>Information of tet(X)-positive strains both from previous studies and the current study were plotted to create a classification figure using Adobe Illustrator v22.1. The map in Figure 1 was created using Edraw Max v9.4.<br>The top unique protein sequences were selected and submitted to the Guidance2 server to evaluate the quality of the alignment and identify potential regions and sequences that reduce the quality of alignment. Multiple sequence alignment was performed using MUSCLE v3.8.31 with default parameters. A model with the least score (JTT+I) evaluated using the Models function in MEGA 7 was selected as the best amino acid substitution model to reconstruct the Maximum-Likehood tree.

For manuscripts utilizing custom algorithms or software that are central to the research but not yet described in published literature, software must be made available to editors and reviewers. We strongly encourage code deposition in a community repository (e.g. GitHub). See the Nature Research guidelines for submitting code & software for further information.

Data

Policy information about availability of data

All manuscripts must include a data availability statement. This statement should provide the following information, where applicable:

- Accession codes, unique identifiers, or web links for publicly available datasets
- A list of figures that have associated raw data
- A description of any restrictions on data availability

The whole genome sequencing data of all strains in this study have been submitted to the NCBI database under the BioProject accession number PRJNA595705 [https://www.ncbi.nlm.nih.gov/bioproject/?term=PRJNA595705]. The 16S rRNA gene sequences of all strains in this study have been deposited in the NCBI GenBank database under accession numbers MT793124 [https://www.ncbi.nlm.nih.gov/nuccore/MT793124]-MT793229 [https://www.ncbi.nlm.nih.gov/nuccore/MT793229].

Field-specific reporting

Please select the one below that is the best fit for your research. If you are not sure, read the appropriate sections before making your selection.

- [x] Life sciences
- [ ] Behavioural & social sciences
- [ ] Ecological, evolutionary & environmental sciences

For a reference copy of the document with all sections, see nature.com/documents/nr-reporting-summary-flat.pdf

Life sciences study design

All studies must disclose on these points even when the disclosure is negative.

Sample size	we screened 1547 K. pneumoniae and 1250 E. coli isolates recovered from clinical samples that were collected from 77 hospitals located in 26 provinces in China during the period 1994-2019. We also screened 3892 clinical Gram-negative bacteria belonging to Bacteroidetes and Proteobacteria from different hospitals in Zhejiang Province. These bacteria include 2591 strains of Acinetobacter spp., 612 S. maltophilia strains, 376 Flavobacteriaceae strains, 136 strains of Burkholderia. spp., 108 strains of Pseudomonas spp. and 69 other Gram-negative isolates collected during the period 1994-2019. No sample size calculation was performed. All bacteria were collected in a nationwide surveillance process , thus the samples are representative of the whole country. The sample size is sufficient to infer the carriage rate of tet(X) in clinical settings as well as the ancestral source of tet(X).
Data exclusions	No data were excluded from analysis
Replication	All qRT-PCR reactions were performed in triplicate. All replication attempts were successful, and the results were presented using mean ± standard deviation. Species identification, antimicrobial resistance testing and transconjugation were performed in triplicate and repeated three times with similar results. All attempts at replication were successful.
Randomization	Randomization was not used since there are no experimental groups.
Blinding	Blinding is not relevant to this study since group allocation is not involved.

Reporting for specific materials, systems and methods

We require information from authors about some types of materials, experimental systems and methods used in many studies. Here, indicate whether each material, system or method listed is relevant to your study. If you are not sure if a list item applies to your research, read the appropriate section before selecting a response.

Materials & experimental systems

n/a	Involved in the study	
x		Antibodies
x		Eukaryotic cell lines
x		Palaeontology and archaeology
x		Animals and other organisms
x		Human research participants
x		Clinical data
x		Dual use research of concern

Methods

n/a	Involved in the study	
x		ChIP-seq
x		Flow cytometry
x		MRI-based neuroimaging
