# Supplementary material for: Epidemiological and phylogenetic analysis reveals Flavobacteriaceae as potential ancestral source of tigecycline resistance gene tet(X)
Source: Nat Commun. 2020 Sep 16;11:4648. doi: 10.1038/s41467-020-18475-9 (PMC7494873; doi:10.1038/s41467-020-18475-9)
Supplement: Supplementary file 4 — Description of Additional Supplementary Files [file 41467_2020_18475_MOESM4_ESM.pdf]

Description of Additional Supplementary Files

File Name: Supplementary Data 1
Description: Source, genomics and antimicrobial resistance profiles of tet(X)-positive bacteria in this study.

File Name: Supplementary Data 2
Description: *tet*(X)-positive bacteria from previous studies.
